# Unilateral renal cortical necrosis

**DOI:** 10.1016/j.radcr.2024.02.108

**Published:** 2024-03-30

**Authors:** Varisha Shahzad, Katherine McDonald, Robert Murphy, Yvonne O'Meara

**Affiliations:** a Department of Nephrology, Mater Misericordiae University Hospital, Dublin, Ireland; b School of Medicine, University College Dublin, Dublin, Ireland

**Keywords:** Renal cortical necrosis, Hydronephrosis, Hydroureter, Pyelonephritis

## Abstract

A 51-year-old woman presented to her local emergency department with acute onset right-sided flank pain and nausea. Her blood results on admission were largely unremarkable aside from leucocytosis and neutrophilia. Two days after admission, she developed the following: stage 3 AKI with oliguria, anaemia, thrombocytopenia, and acute derangement of liver function tests. A computed tomography of the kidney ureter bladder demonstrated a right-sided 4 mm obstructing vesicoureteric junction stone with associated hydronephrosis and hydroureter. She was transferred to a tertiary care centre; gram negative sepsis was confirmed with a Proteus on blood culture and laboratory findings were in keeping with DIC.

She was treated with Tazobactam/Piperacillin and intravenous fluids. In addition, further imaging showed improving right-sided hydronephrosis and left renal cortical necrosis. The aetiology of this presentation was sepsis complicated by disseminated intravascular coagulation. The coagulopathy likely contributed to the unilateral renal cortical necrosis.

Cortical necrosis usually affects both kidneys, and is typically a complication of sepsis, shock, or obstetrical trauma. To our knowledge, there are only 2 reported cases of unilateral renal cortical necrosis and contralateral hydronephrosis with renal colic and septic shock. Potential pathogenetic mechanisms are discussed.

## Introduction

Cortical necrosis clinically presents as a rapid reduction in glomerular filtration rate (GFR) accompanied by oliguria. In the native kidney, cortical necrosis can be caused by thrombosis of interlobular or larger arteries, massive cholesterol emboli, septic abortion, or other obstetrical complications. Other contributing factors include vascular spasm, microvascular injury, or intravascular coagulation. Cortical necrosis normally affects both kidneys, however, here we describe a case of unilateral cortical necrosis [1].

## Case presentation

**Fig. 1:**
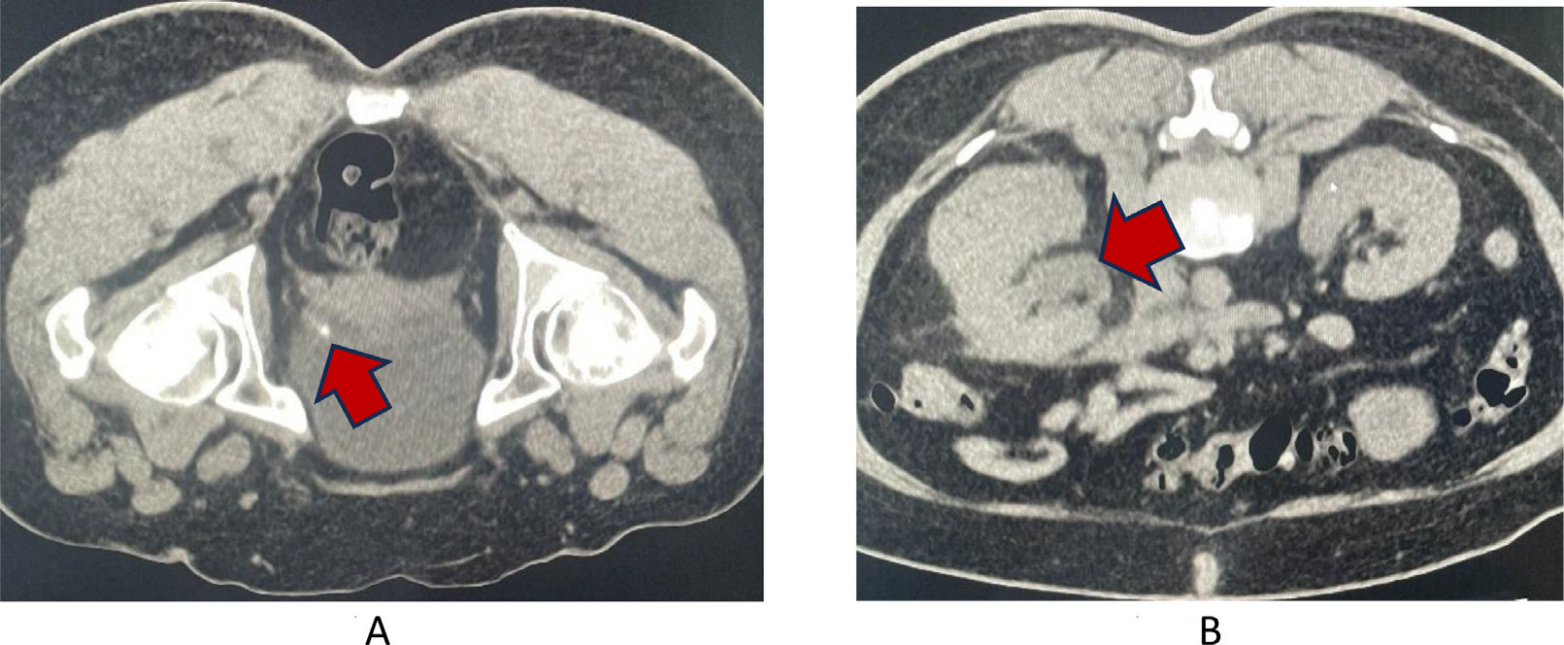
Imaging modality: noncontrast enhanced computed tomography. Findings: (A) a 4 mm obstructing stone at the level of the right VUJ (red arrow). (B) Proximal hydronephrosis and hydroureter as well as some mild enhancement of the right renal pelvis and distal ureter (red arrow).

**Fig. 2:**
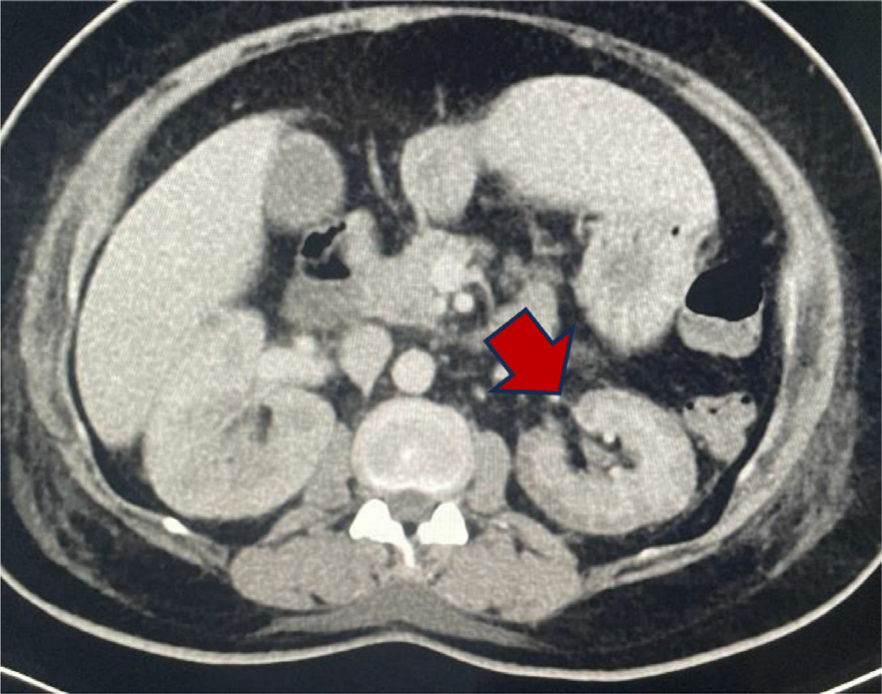
Imaging modality: contrast enhanced computed tomography of the abdomen. Findings: There is diffuse cortical hypoenhancement of the left renal cortex with normal enhancement of the medulla. This is due to renal cortical necrosis.

We report the case of a 51-year-old female who had returned from a trip to Thailand, throughout which she had been well and had no sick contacts. On the day of her return to Ireland, she developed right-sided flank pain and nausea. She had a past medical history of hypertension and gastritis. She attended her local emergency department, where a computed tomography of the kidney ureter bladder (CT-KUB) was ordered. This demonstrated a right-sided 4 mm obstructing vesicoureteric junction (VUJ) stone with associated hydronephrosis and hydroureter (Fig. 1, Fig. 2).

Her blood results upon presentation were as follows urea 4.81 mmol/L, creatinine 60 μmol/L, sodium 139 mmol/L, potassium 3.4 mmol/L, haemoglobin 14.0 g/dL, white cell count 14.73 × 10^9^, neutrophils 13.81 × 10^9,^ platelets 296 × 10^9^ and c-reactive protein (CRP) 7.

While awaiting transfer to a tertiary centre under Urology for further management of the stone, the patient's renal function suddenly deteriorated. She became oliguric with a urine output of 100 mL over 6 hours despite receiving furosemide 40 mg intravenously. In addition, platelets dropped to 36 × 10^9,^ liver function tests became acutely deranged, and CRP increased. She was transferred to the high dependency unit of the accepting tertiary centre. Her kidney function continued to deteriorate (Fig. 1A). Given the acute deterioration, a repeat CT with contrast (Fig. 1B) was requested which showed a 4 mm obstructing stone at the level of the right VUJ with proximal hydronephrosis and hydroureter and features consistent with pyelonephritis.

There was diffuse cortical hypoenhancement of the left renal cortex with normal enhancement of the medulla in keeping with a “reverse rim sign” of cortical necrosis (Fig. 2).

Given the patient's deranged coagulation profile, advice from Haematology was sought. The patient's coagulation profile was as follows; platelets 21 × 10^9^/L, d-dimer >20 mg/L, fibrinogen 4.79 g/L, APTT 28.7 seconds, prothrombin time 15.3 seconds. Initially, atypical haemolytic uraemic syndrome or thrombotic microangiopathy was considered. However, when her blood cultures confirmed a Proteus bacteraemia, a diagnosis of urosepsis complicated by disseminated intravascular coagulation (DIC) was made. The coagulopathy likely contributed to the unilateral renal cortical necrosis, of which only a handful of cases have been reported.

The patient was reviewed by Urology, who recommended a conservative approach and a repeat the CT-KUB within 48 hours. The repeat CT renal showed that the obstructing stone had migrated into the bladder with resolving hydronephrosis and hydroureter.

**Fig. 3:**
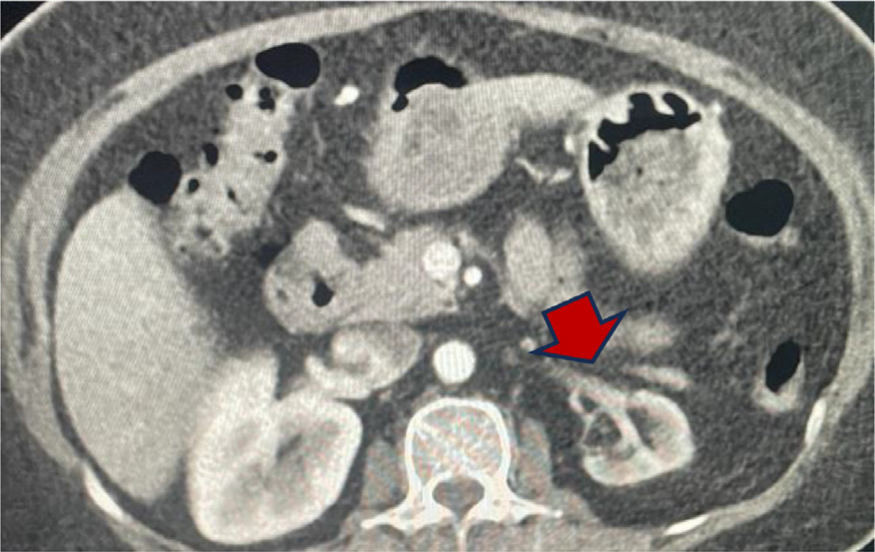
Imaging modality: contrast enhanced computed tomography. Findings: Renal atrophy (red arrow).

During her inpatient stay, she responded well to intravenous antibiotics (Tazobactam/Piperacillin), and her inflammatory markers decreased. In addition, her liver function tests, and renal function tests improved. She was discharged from the hospital and followed up in the Nephrology outpatient service. Her renal function tests had returned to baseline; however, the right kidney was found to be atrophic on follow up imaging (Fig. 3).

## Discussion

Two types of cortical necrosis have been described based on histology, 1 being diffuse cortical necrosis whereby there is confluent global cortical destruction which extends into the columns of Bertin. The thin rim of subcapsular and Juxtamedullary tissue remains preserved. The outcome of diffuse cortical necrosis is often irreversible renal failure. The second type of cortical necrosis is patchy as in this case where there is a contiguous area of cortical necrosis involving up to one-third to half of the entire cortical tissue. As in other cases of patchy cortical necrosis there was partial recovery of renal function in this case [2].

Usually, the first modality of imaging is an ultrasound, acute cortical necrosis will demonstrate a hypoechoic renal cortex [3] while in a pyelonephritis the kidneys may be enlarged and have a hypoechoic parenchyma with loss of the normal corticomedullary junction [4].

Ifregan et al. described the following radiological findings of cortical necrosis; hypodensity of the renal cortex, hyper density of the medulla, and lack of excretion of contrast into the collecting system [5]. In arterial phase the CT scan shows enhancement of the interlobular and arcuate arteries adjacent to the nonenhancing cortex. In the portovenous phase, the CT shows enhancement of the renal medulla with a hypoattenuating, nonenhancing cortex; this is called the “reverse rim sign” [6]. A CT in case of a pyelonephritis can demonstrate several areas to be affected, and in majority of cases the lesions are well-defined, wedge-shaped areas with their bases at the periphery and apexes towards the renal sinus, with the renal parenchyma sometimes demonstrating a striated appearance. These findings indicate hypoperfusion secondary to arteriolar vasoconstriction and inflammatory response [7].

If an MRI is carried out it may display a low signal intensity on both T1 and T2 weighted sequences affecting the inner renal cortex and the columns of Bertin [8] while a pyelonephritis, depending on the stage of infection will show enlargement of the kidney, thickened parenchyma, and an impairment of corticomedullary differentiation [9].

The unilateral form of cortical necrosis is rare. There have been 2 previous reported cases of unilateral renal cortical necrosis in association with renal colic and sepsis. Quin et al. described a similar presentation of unilateral cortical necrosis and contralateral hydronephrosis with renal colic and septic shock [10]. Vadala et al. described a patient with ARDS and severe sepsis who also had left sided hydroureteronephrosis secondary to a calculus with right cortical necrosis [11]. The authors hypothesized the necrosis to be ischemic, secondary to diffuse thrombotic microangiopathy promoted by disseminated intravascular coagulation.

Most cases of unilateral cortical necrosis describe preserved renal function in the contralateral kidney, usually associated with hydronephrosis. There are 2 theories regarding how the contralateral hydronephrosis helps to conserve kidney function. Almost 50 years ago, Watchi and Altman described how prolonged ureteral obstruction reduces renal blood flow; this could somehow reduce the extent of glomerular thrombosis [12]. In addition, it has been reported that hydronephrosis alters glomerular hemodynamic by opening anatomical shunts, thus possibly explaining the complete absence of thrombi [13]. Secondly, hydronephrosis may increase the production of agents such as insulin-like growth factor (IGF-1), which may offer protection to the renal architecture or prostaglandin E2 (PGE2), which counteracts vasoconstrictive responses of afferent arterioles, thereby modulating the actions of angiotensin II, in turn providing protection against renal ischemia [14,15].

Bilateral cortical necrosis is usually associated with poor outcomes. The above theories may explain how our patient had relatively conserved renal function while having unilateral cortical necrosis and contralateral hydronephrosis.

## Patient consent

Informed consent was obtained from the patient.
